# Total Synthesis of Bioactive Marine Meroterpenoids: The Cases of Liphagal and Frondosin B

**DOI:** 10.3390/md16040115

**Published:** 2018-03-31

**Authors:** Yan Zong, Weijia Wang, Tao Xu

**Affiliations:** 1 Key Laboratory of Marine Drugs, Ministry of Education, School of Medicine and Pharmacy, Ocean University of China, 5 Yushan Road, Qingdao 266003, China; 17864275115@163.com (Y.Z.); weijia15764218251@126.com (W.W.); 2 Laboratory for Marine Drugs and Bioproducts of Qingdao, National Laboratory for Marine Science and Technology (QNLM), 1 Wenhai Road, Qingdao 266237, China

**Keywords:** natural product synthesis, total synthesis, liphagal, frondosin

## Abstract

Liphagal and frondosin B are two marine-derived secondary metabolites sharing a very similar polyfused-benzofuran skeleton. The two tetracyclic meroterpenoids were isolated from marine sponges, both featuring a 6-5-7-6 fused ring system. A preliminary bioactive study shows that (+)-liphagal is a selective kinase (PI3K α) inhibitor, while (+)-frondosin B is shown to inhibit the binding of the cytokine interleukin-8 (IL-8) to its receptor, CX-CLR1/2. The unique structures and interesting biological profiles of these two meroterpenoids have attracted considerable attention from synthetic chemists. Herein we summarize the synthetic efforts with respect to (+)-liphagal and (+)-frondosin B during the past two decades.

## 1. Introduction

**Figure 1:**
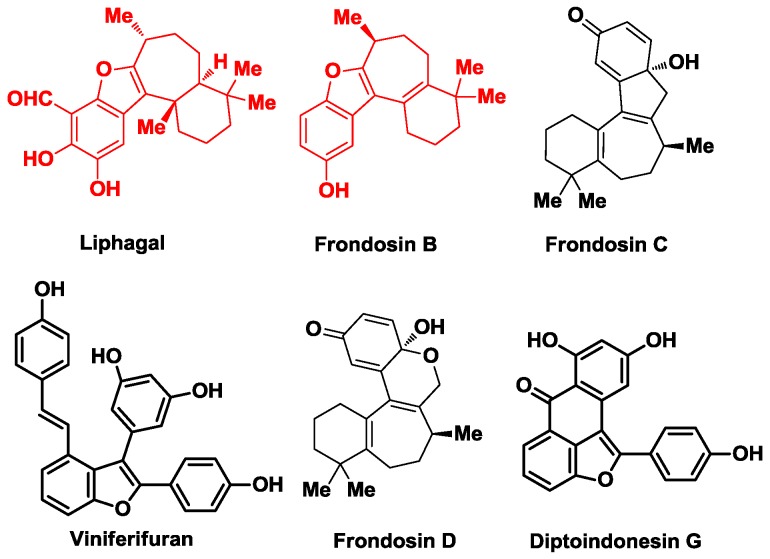
Representative meroterpenoids of marine-derived natural products.

Climate change is placing great pressure on the ocean’s ecosystem, exacerbating the evolutionary pressure on all the creatures it harbors [1]. Evolution is based on and demonstrated by numerous delicate gene mutations from a biological standpoint, and also directly leads to vast numbers of newly transcripted proteins, which are rooted in the mutated genes. These proteins, together with existing ones, constitute a biosynthetical toolbox for the generation of many functional molecules. Following the above logic, one of the consequences of climate change is the generation of a miraculous diversity of organic molecules with varying degrees of complexity. Modern molecular biology tells the whole picture of life on a molecular basis, shedding lights on studies of marine natural products, especially those termed as secondary metabolites [2]. The great abundance of species, as well as purposely biosynthesized small molecules, constitute humankind’s greatest opportunity for drug identification. The ocean is a unique and excellent source of intriguing natural products with significant biological activities. In recent times, more and more novel marine natural products have been isolated and developed for pharmaceutical agents [3,4]. Among them, two secondary metabolites containing polyfused benzofuran skeletons, namely liphagal and frondosin B, have drawn great attention from synthetic chemists world-wide (Figure 1). (+)-Liphagal and frondosin B are two marine tetracyclic meroterpenoids isolated from marine sponges, both featuring a 6-5-7-6 fused ring system. A preliminary bioactive study shows that (+)-liphagal is a selective kinase (PI3K α) inhibitor [5], while (+)-frondosin B is shown to inhibit the binding of the cytokine interleukin-8 (IL-8) to its receptor, CX-CLR1/2 [6,7,8]. The unique structures and interesting biological profiles of these two meroterpenoids have attracted considerable attention from synthetic chemists. Herein we summarized the synthetic efforts towards (+)-liphagal and (+)-frondosin B.

(+)-Liphagal was first isolated in Dominica from the marine sponge *coralliphaga* by professor Andersen and coworkers in 2006 [5]. The primary fluorescent polarization enzyme assay showed that liphagal inhibited PI3K-α with an IC_50_ value of 100 nM, and was approximately 10-fold more potent against PI3K-α than PI3K-γ. In vitro cell assays showed that liphagal was cytotoxic to the LoVo (human colon: IC_50_ = 0.58 µM), CaCo (human colon: IC_50_ = 0.67 µM), and MDA-468 (human breast: IC_50_ = 1.58 µM) tumor cell lines. These preliminary biological studies revealed the great potential of liphagal to be developed into new generations of kinase inhibitor and anti-tumor drugs.

## 2. The Biosynthetic Proposal for Liphagal

**Figure 2:**
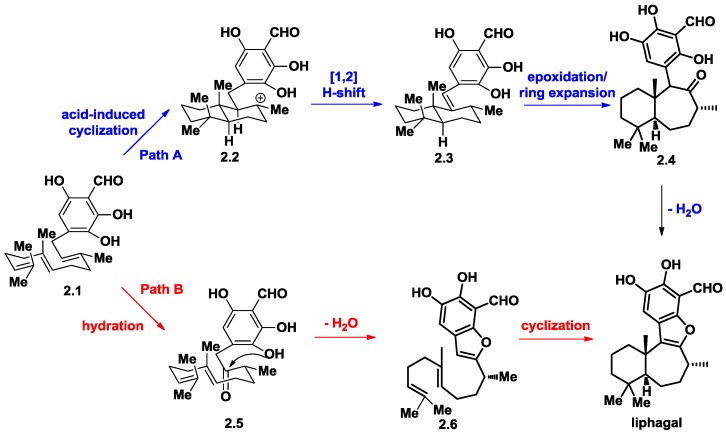
Proposed biosynthesis of liphagal.

Andersen and co-workers proposed two possible biosynthetic routes towards liphagal from farnesylated trihydroxybenzaldehyde (**2.1**), namely path A and path B, as shown in Figure 2. Path A involves a proton-induced polyene cyclization to give a transfused decalin skeleton (**2.2**), followed by a [1,2] H-shift to obtain siphonodictyal B (**2.3**), a natural product previously reported by Faulkner and co-workers [6,7]. Siphonodictyal B could be converted to a “liphagane” core bearing a 6-5-7-6 fused ring system (**2.4**) via epoxidation and ring expansion. Following ketoformation/dehydration, liphagal would be furnished. Other the other hand, path b outlines a more direct route to liphagal that starts with the conversion of **2.1** to the ketone **2.5** via region- and diastereo-selective hydration. Then, benzofuran (**2.6**) could be generated after dehydration. Proton-initiated cyclization would lead directly to liphagal. The latter route suggests an efficient synthetic route to the biomimetic total synthesis of liphagal.

## 3. Total Synthesis of Liphagal

Liphagal has attracted attention from groups with interests in synthetic products all over the world since its isolation in 2006. Overall, five total syntheses and one formal synthesis have been reported, not to mention many attempts. Herein, we summarize all the methods for total and formal synthesis of liphagal in a chronological sequence, and discuss them in detail, focusing on transformation rather than strategy.

### 3.1. Biomimetic Total Synthesis of (+)-Liphagal

**Figure 3:**
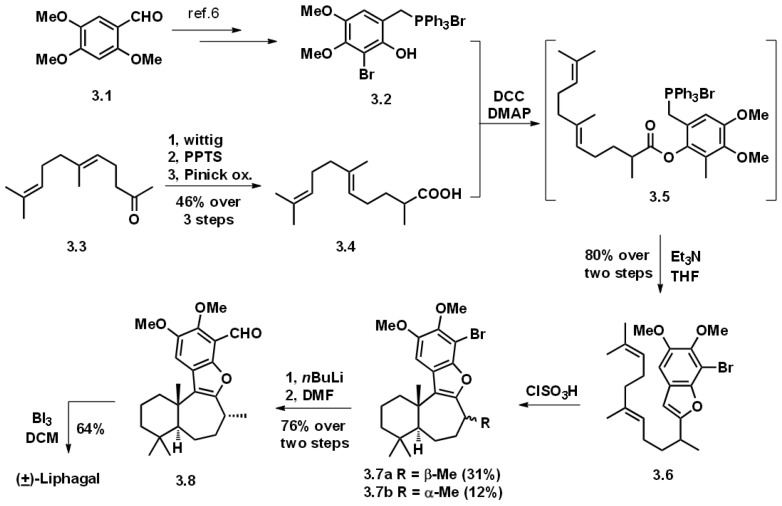
Biomimetic synthesis of liphagal by Andersen and co-workers.

Inspired by the biogenetic routes to liphagal, Andersen accomplished the total synthesis of liphagal following a biomimetic strategy (Figure 3). The synthesis of racemic liphagal started from commercially available 2,4,5-trimethoxybenzaldehyde (**3.1**), through an expeditious six-step routine transformation to achieve phosphonium salt (**3.2**). The other coupling fragment (**3.4**) was synthesized from geranylacetone (**3.3**) via Wittig and oxidation adjustment, with an overall 46% yield.

Coupling of the two fragments was realized under standard DCC condensation condition. The resulting product (**3.5)** was not purified but directly treated with a trimethylamine-affording benzofuran product (**3.6**) in a gratifying 80% yield. The biomimetic cyclization conditions were screened and it was found that ClSO_3_H would induce moderately effective polyene cyclization, obtaining a pair of C_8_-diastereoisomers with the desired isomer **3.7a** as major product. The success of this key transformation also supported the authors’ biosynthetic proposal. The first total synthesis of racemic liphagal was achieved via halogen–lithium-initiated formylation and selective deprotection using three steps. Andersen’s seminal synthesis on liphagal greatly inspired the following synthetic studies towards it.

### 3.2. Mehta’s Formal Synthesis of (+)-Liphagal

**Figure 4:**
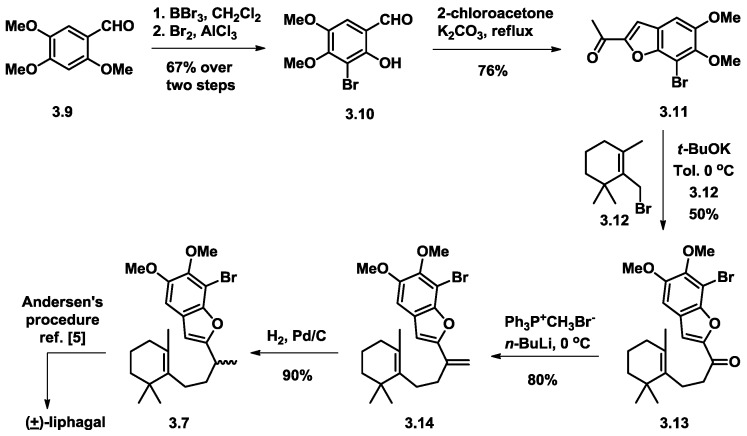
Mehta’s formal synthesis of liphagal.

Mehta and co-workers [9] reported a nine-step formal total synthesis towards (+)-liphagal from a commercial starting material. This was a scalable and diversity-oriented approach. Similar to Anderson’s work, from aldehyde (**3.9**), the benzofuran compound (**3.11**) was synthesized from 2-chloroacetone and brominated aldehyde (**3.10**) in a basic condition, which was coupled with allylic bromide (**3.12**) via neucleophilic substitution, affording cyclization precursor **3.14** upon Wittig olefination and hydrogenation. The cyclization step was following Andersen’s seminal work and formal synthesis of (+)-liphagal was achieved (Figure 4).

### 3.3. George’s Total Synthesis of (+)-Liphagal

**Figure 5:**
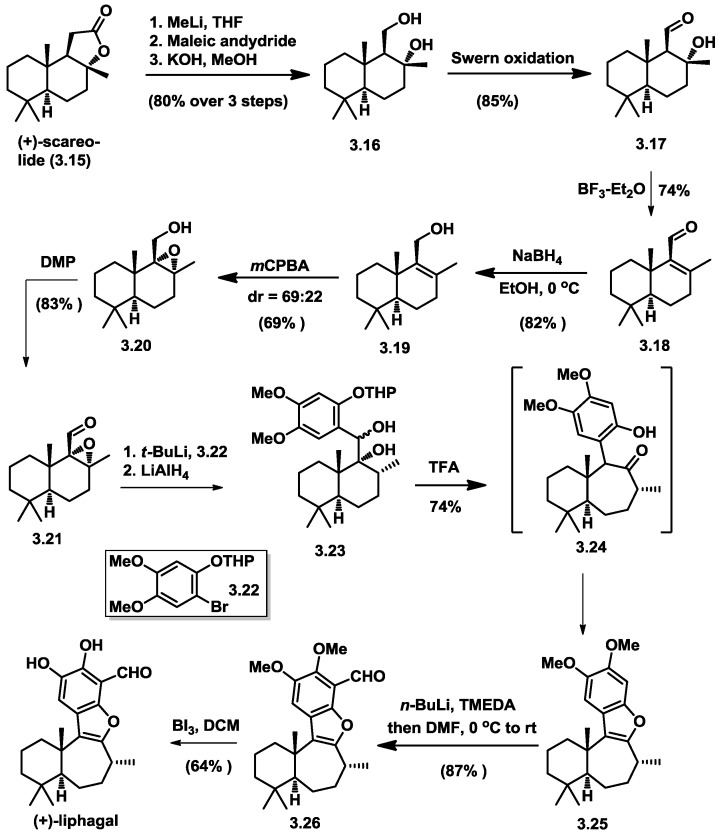
George’s asymmetric total synthesis of liphagal.

An asymmetric total synthesis of liphagal was first reported by Geogre and co-workers based on a chiral-pool strategy. The synthesis began with commercially readily available (+)-sclareolide and took about 13 synthetic steps with 9% overall yield [10] to obtain (+)-liphagal. The key step involved a pinacol rearrangement and a benzofuran formation cascade. (Figure 5).

Starting with (+)-sclareolide (**3.15**), chiral 1,3-diol (**3.16**) was obtained in three steps with an overall 80% yield. Upon swern oxidation, the resulting β-hydroxy aldehyde (**3.17**) was dehydrated with BF_3_-Et_2_O to an α,β-unsaturated aldehyde (**3.18**). Reduction of the aldehyde with NaBH_4_ provided allylic alcohol (**3.19**), which was subjected to m-CPBA mediated epoxidation condition affording a pair of epoxides (**3.20**) as a 3:1 mixture (separable by SiO_2_ flash column chromatography). An oxidation state adjustment with Dess–Martin reagent lead to the 1,2-addition precursor **3.21**. The aryllithium fragment was generated through lithium–halogen exchange from arylbromide (**3.22**) and *t*-BuLi. A LiAlH_4_-mediated regioselective expoxide opening successfully occurred, affording 1,2-diol (**3.23**), presumably due to the benzylic alcohol’s coordination, as well as steric hinderance of the angular Me group. The key pinacol rearrangement was carried out diastereoselectively by treatment of 1,2-diol (**3.23)** with TFA in DCM, providing 6,7-fused ring cycle **3.24** as a transient product. The subsequent ketoformation/dehydration occurred automatically, thus affording a 6-5-7-6 ring system in a 74% yield. Installation of the formyl group was started with ortholithiation, followed by quenching with DMF. (+)-Liphagal was achieved upon the previously described demethylation.

### 3.4. Manzaneda’s Total Synthesis of (+)-Liphagal

**Figure 6:**
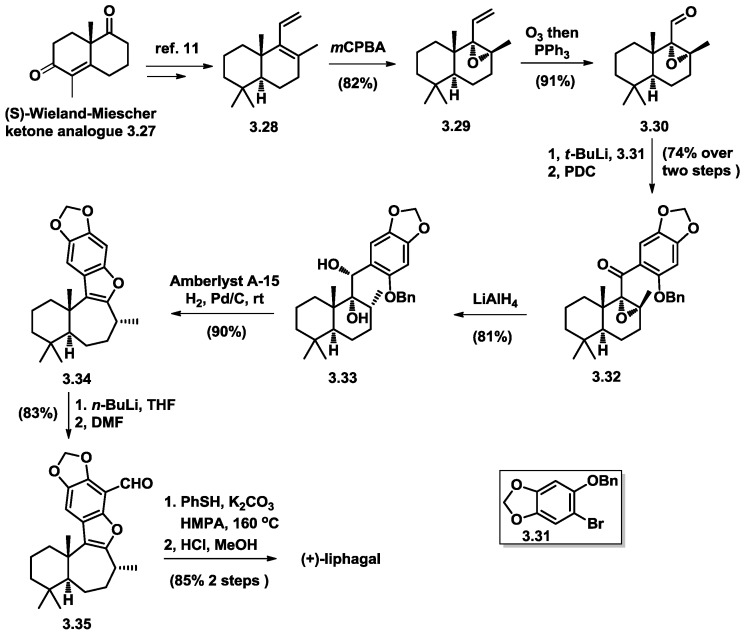
Manzaneda’s asymmetric total synthesis of liphagal.

Manzaneda and co-workers started their enantioselective total synthesis [11] of (+)-liphagal with a previously reported advanced diene compound (**3.28**) [12], which was prepared from an (S)-Wieland–Mischer ketone analogue (**3.27**) (Figure 6). Substrate-controlled regio- as well as diastereo-selective epoxidation with m-CPBA afforded mono-epoxide **3.29**, which upon ozonolysis, led to aldehyde (**3.30**). 1,2-addition of aldehyde (**3.30**) with aryllithium, generated from arylbromide (**3.31**) and *t*-BuLi, followed by PDC-mediated oxidation yielded tetracycle intermediate **3.32**. The following synthetic transformations were very similar, as described previously. The key tetracyclic “liphagane” skeleton (**3.34**) is formed in one step after the hydrogenation of a dihydroxyl drimanephenol benzyl ether (**3.33**) in the presence of cationic resin. The total synthesis was achieved in 12 steps.

### 3.5. Stoltz’s Total Synthesis of (+)-Liphagal

**Figure 7:**
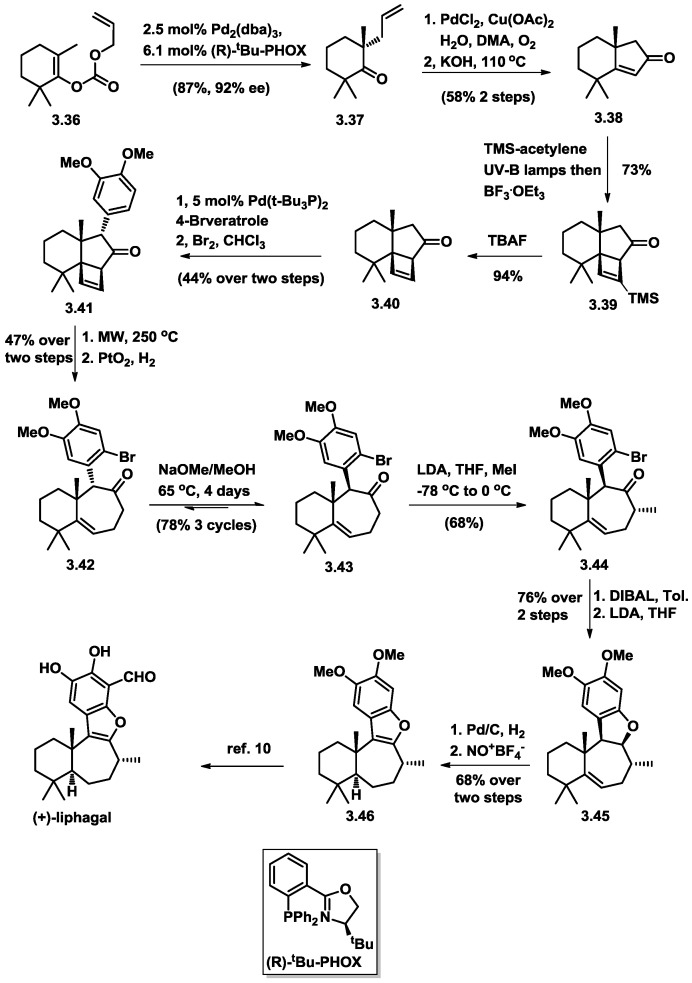
Stoltz’s total synthesis of (+)-liphagal.

In 2011, Stoltz and co-workers successfully completed the first catalytic asymmetric total synthesis of (+)-liphagal [13] in 19 steps from commercially available starting materials. The key reactions were catalytic enantioselective alkylation and pericyclic cyclobutene-ring opening (Figure 7).

The forward synthesis commenced with carbonate (**3.36)** using their previously reported palladium-catalyzed decarboxylative alkylation methodology [14]. The tetrasubstituted ketone **3.37** was obtained in 87% yield and 92% ee. Following their previously investigated two-step sequence (which involved a Wacker oxidation and a Robinson annulation), the desired 6-5 fused enone compound **3.38** was obtained. The synthesis continued with exposure of enone to trimethylsilylacetylene under UV irradiation, which promoted a [2+2] photocycloaddition. Exposure of the crude reaction mixture to BF_3_·OEt_2_ resulted in the formation of a single silylated cyclobutene product (**3.39)**. Subsequent removal of the trimethylsilyl group with TBAF and then microwave assisted palladium-catalyzed α-arylation with 4-bromoveratrole installed the electron-rich aromatic moiety, which was regioselectively brominated with Br_2_, affording cyclobutene (**3.41)**. An optimized [15] heat-induced pericyclic ring opening reaction took place smoothly, followed by Adams catalyst-promoted selective 1,4 conjugate addition under a hydrogen atmosphere. The desired 6-7 fused ring skeleton (**3.42**) was obtained and epimerization of the aryl substituent in a 78% overall yield was accomplished after three cycles of equilibration, providing the desired configuration in precursor **3.43** for the following diastereoselective α-methylation. Reduction of the hindered ketone with DIBAL produced alcohol, and then the highly congested dihydrobenzofuran product **3.45** was obtained in the presence of LDA via an aryne intermediate.

Diastereo-hydrogenation of the trisubstituted alkene using Pd/C in ethanol led to exclusive formation of the 6,7-trans fused ring structure. The benzofuran moiety in **3.46** was formed by employing a unusual oxidation condition with nitrosonium tetrafluoroborate through hydride abstraction. After installation of an aldehyde group and demethylation, the enantioselective total synthesis of (+)-liphagal was completed.

### 3.6. Katol’s Total Synthesis of (+)-Liphagal

**Figure 8:**
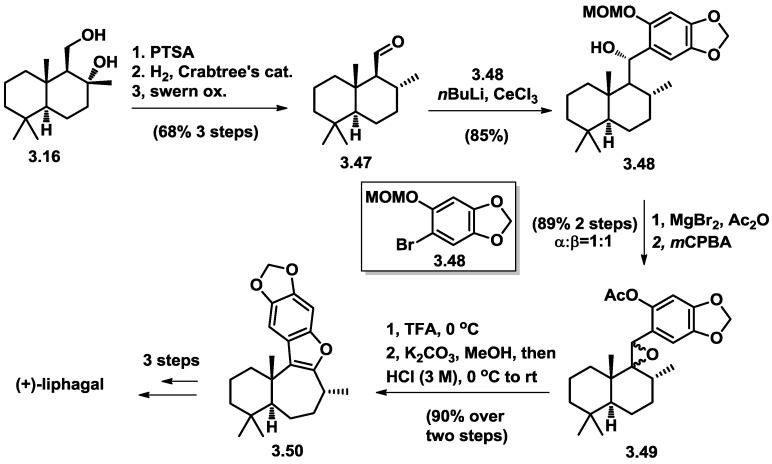
Katoh’s total synthesis of (+)-liphagal.

In 2014, Katoh and co-workers [16] reported a biomimetic total synthesis of (+)-liphagal in 29% overall yield in 13 steps in the longest linear trajectory from (+)-sclareolide (Figure 8). The key reaction also features the afore-mentioned pinacol rearrangement from epoxide. 

### 3.7. Synthetic Studies towards Liphagal

By the end of 2017, there were four reports on the synthetic efforts towards liphagal and/or its analogues [17,18,19,20,21,22,23]. As this review only summarizes the total synthesis or formal total synthesis of liphagal, these reports will not be discussed here in detail. 

## 4. Total Synthesis of Frondosin B

Sharing its structural similarity with liphagal but with fewer chirality centers, frondosin B has been viewed as “star molecule” ever since its isolation in 1997 from a marine sponge identified as *Dysidea frondosa* [8]. Frondosin B, together with its family members (frondosin A–E) has been reported as an inhibitor of cytokine interleukin-8 (IL-8), potentially acting as a druggable target protein. The frondosins features a bicyclo[5.4.0]undecane core with several structural variants decorated by annulation types. Frondosin B’s unconfirmed stereocenter, as well as its biological profile, has attracted total synthetic chemists world-wide. Herein, we describe and discuss seven asymmetric total synthesis of (+)-frondosin B and several other synthetic efforts briefly.

### 4.1. Danishefsky’s Total Synthesis of (+)-Frondosin B

In 2001, Danishefsky reported the first asymmetric total synthesis of (+)-frondosin B in 17 total steps with an approximately 1% overall yield [24,25]. The synthesis introduced chirality by way of Sharpless epoxidation of known materials. Furthermore, Danishefsky’s synthesis established that (+)-frondosin B(2) contains an R stereocenter at C8 through correlation with the reported optical rotation.

**Figure 9:**
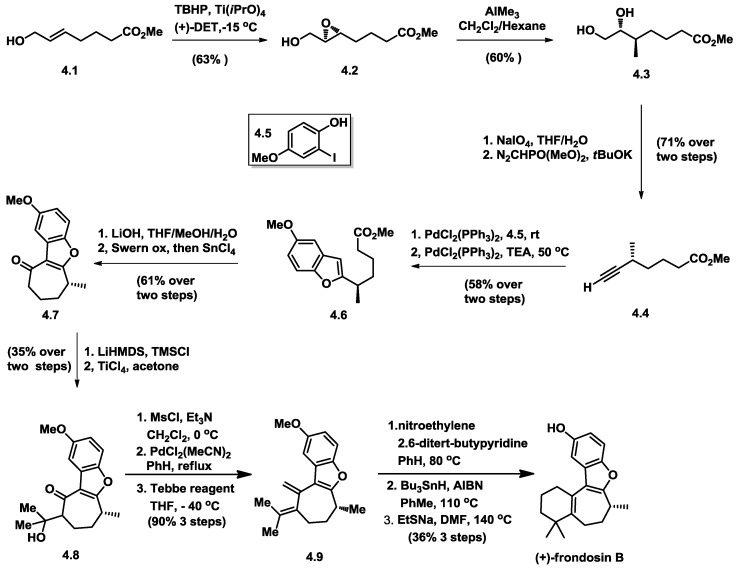
Danishefsky’s total synthesis of (+)-frondosin B.

Danishefsky’s synthesis commenced with a Sharpless epoxidation of the known methyl heptenoate (**4.1**) to give the known (−)-methyl (5S,6S)-5,6-epoxy-7-hydroxyheptanoate (**4.2**) (Figure 9). Epoxide opening with application of excess AlMe_3_ at −78 °C provided (+)-methyl (5R,6R)-6,7-dihydroxy-5-methylheptanoate (**4.3**), establishing the single stereogenic center of C8 chirality in frondosin B. Cleavage of the resulting diol **4.3** with NaIO_4_ afforded aldehyde, which was then treated with Seyferth–Gilberts reagent, yielding terminal alkyne **4.4** in 71% yield. The benzofuran compound was in good yield through Sonogashira coupling over two steps. Through saponification of the resulting ester and subsequent conversion into the corresponding acyl chloride, the key cycloheptenone was produced through an intramolecular Friedel–Crafts acylation, promoted by tin(IV) chloride.

To avoid epimerization of the C8 center, the Mukaiyama aldol reaction was utilized to generate tertiary alcohol **4.8**, which was converted to the mesylate. Elimination followed, providing a ca. 1:1 mixture of conjugated and non-conjugated olefinic isomers. Olefin isomerization was conducted by heating the olefinic mixture at reflux with PdCl_2_(MeCN)_2_ in benzene without racemization of the C8 carbon center. Methylenation of the conjugated ketone with Tebbe’s reagent yielded **4.9**.

Diels–Alder cyclization was conducted using electron-deficient nitroethylene under acid-free conditions. The nitro group can be easily removed under free radical conditions. Finally, deprotection with sodium ethanethiolate produced (+)-frondosin B in high yield. The optical rotation value ([α]_D_ = +15.2) is very close to that of the naturally occurring sample ([α]_D_ = +18.5), and thus it was concluded that (+)-frondosin B exists naturally as the R-configuration. 

### 4.2. Trauner’s Total Synthesis of (−)-Frondosin B

**Figure 10:**
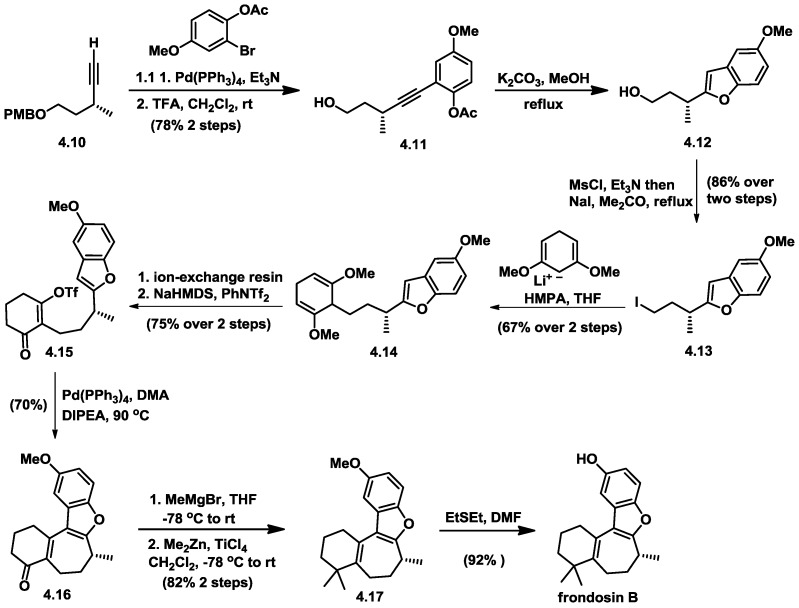
Trauner’s total synthesis of (−)-frondosin B.

Trauner’s synthesis [26,27] commenced with R-configured alkyne **4.10**, which was synthesized in a similar protocol as Danishefsky’s [22] (Figure 10). Aryl bromide was used as a Sonogashira coupling partner to produce the desired arene, which after acidic removal of the primary p-methoxybenzyl group yielded alcohol **4.11**. Treatment of alcohol **4.11** with K_2_CO_3_ in methanol enabled saponification of the phenolic acetate and concomitant cyclization to afford benzofuran (**4.12**) in one step. Alkylation of iodide (**4.13**) with dimethoxylithiocyclohexadiene via an SN_2_ neucleophilic attack lead to the formation of the 1,4-diene compound (**4.14**). A very mild hydrolysis methodology employing an ion-exchange resin was used to cleave the methyl ethers in **4.14**, and the resulting diketone was immediately converted into enol triflate (**4.15**). Treatment of this compound with a catalytic amount of Pd(PPh_3_)_4_ and Hunig’s base at 90 °C resulted in the formation of the key tetracyclic ring system through the intramolecular Heck reaction. The carbonyl group of **4.16** was successfully converted to a geminal dimethyl group with MeMgBr. Finally, demethylation was performed according to Danishefsky’s previously reported procedure, and frondosin B was obtained enantiomerically. However the observed optical rotation corresponded to the opposite value of the natural product, which is [α]_D_ = –16.8. 

Trauner’s group started their syntheses with an R-configured alkyne stereocenter, but the optical rotation of their synthetic product was opposite to that reported for the natural material. Trauner and co-workers had proposed, according to their work [27], that the structure of frondosin B should actually be reassigned as having the S-configuration. In view of the discrepancy surrounding the absolute configuration at the C8 stereocenter, Trauner proposed that the Danishefsky route must have undergone an inversion of configuration at an early stage of the synthesis, very likely during the nucleophilic opening of epoxy alcohol with AlMe_3_.

This discrepancy in the absolute configuration of (+)-frondosin B sparked a series of syntheses of frondosin marine terpenes. 

### 4.3. Ovaska’s Total Synthesis of (−)-Frondosin B

**Figure 11:**
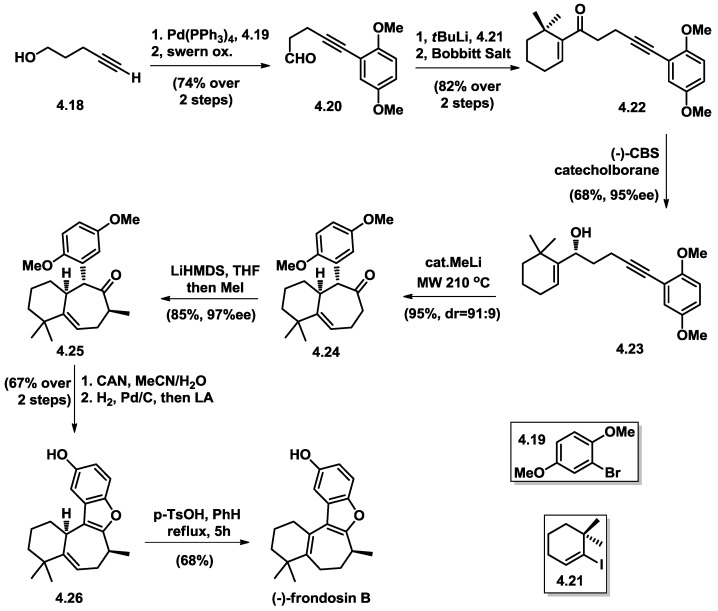
Ovaska’s total synthesis of (−)-frondosin B.

Following the two aforementioned pioneering works, Ovaska and co-workers reported [28] their synthesis of (−)-frondosin B featuring a 5-exo-dig cyclization/Claisen rearrangement from an optically active homopropargylic allylic alcohol (**4.23**) (Figure 11). 

The authors began their synthesis by coupling of the requisite aryl fragment **4.19** to alkyne **4.18** through Sonogashira coupling. After Swern oxidation, followed by 1,2-addition/re-oxidation, the conjugated cyclohexenone **4.22** was obtained in a very efficient way. CBS-mediated enantioselective reduction yielded an optically active (98% ee) allyl alcohol (**4.23**), which was subjected to a cascade transformation. The authors proposed that the mechanism of this cascade reaction started with 5-exo-dig cyclization of OH leading to the formation of 2-olefinic tetrahydrofuran intermediate, which readily underwent a microwave accelerated Claisen rearrangement. This key transformation directly constructed 6,7-fused bicyclic compound **4.24** with good diastereo- as well as enantio-selectivity. The C8 Me group was installed with diastereoselective methylation. The benzofuran moiety in **4.26** was constructed through Lewis acid-induced dihydroquinone, which was generated in situ with Pd/C under an H_2_ balloon after CAN oxidation. Finally, treatment of compound **4.26** with catalytic p-TsOH resulted in the desired olefin isomerization and isolation of (−)-frondosin B in 68% yield. The optical rotation value of this syntheized sample is [α]_D_ = –17.3, which added support for Danishefsky’s initial assignment of the R-configuration to naturally occurring (+)-frondosin B.

### 4.4. MacMillan’s Total Synthesis of (+)-Frondosin B

**Figure 12:**
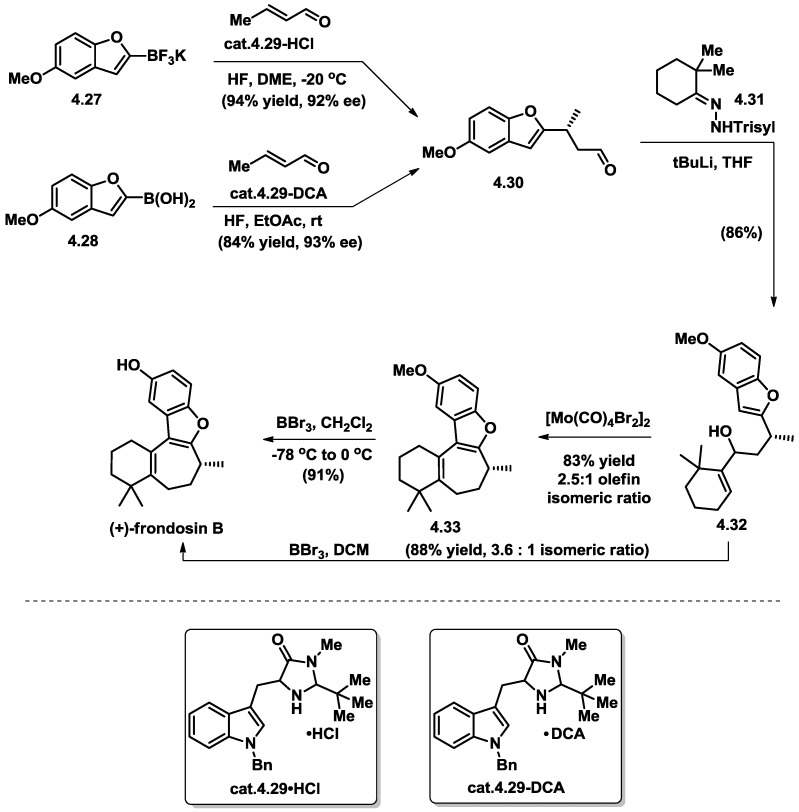
MacMillan’s total synthesis of (+)-frondosin B.

MacMillan and coworkers reported their highly concise total synthesis of (+)-frondosin B in 2010 (Figure 12) [29]. The high efficiency of their synthesis relied on the enantioselective conjugate addition catalyzed by employing their chiral amine catalyst. The synthesis started with 1,4-Michael addition with either heteroaryl trifluoroborate salt (**4.27**) [26] or benzofuran boronic acid (**4.28**) with α,β-unsaturated iminium ion, which was formed in situ between a chiral secondary amine catalyst (**4.29**) and crotonaldehyde. An optically enriched aldehyde (**4.30**) was obtained in 84% yield with 93% ee. Then, a Shapiro reaction with trisylhydrazone (**4.31**) afforded allylic alcohol **4.32** as a mixture of diastereomers in 86% yield. The desired 6-5-7-6 tetracyclic skeleton (**4.33**) was obtained in the presence of catalytic amounts of molybdenum(II) dimer [Mo(CO)_4_Br_2_]_2_ with a 2.5:1 ratio of olefin regioisomers. Upon deprotection of the methyl ether with BBr_3_, the completion of the total synthesis of (+)-frondosin B was accomplished ([α]_D_ = +16.3). In the meantime, it was found that a one-pot transformation starting from alcohol **4.32** through cyclization/deprotection using 3.5 equivalents of BBr_3_ at low temperature would provide (+)-frondosin B along with its olefin isomer in a 3.6:1 ratio in favor of the natural product.

MacMillan and co-workers were able to duplicate early intermediates from both Danishefsky’s and Trauner’s syntheses and showed that although both had initially been based on the R-configuration at C8, a late-stage stereochemical inversion must have occurred in the Trauner’s synthesis, presumably the key intramolecular aryl Heck reaction. This report finalized the C8 stereochemistry debate of past years.

### 4.5. Wright’s Total Synthesis of (−)-Frondosin B

**Figure 13:**
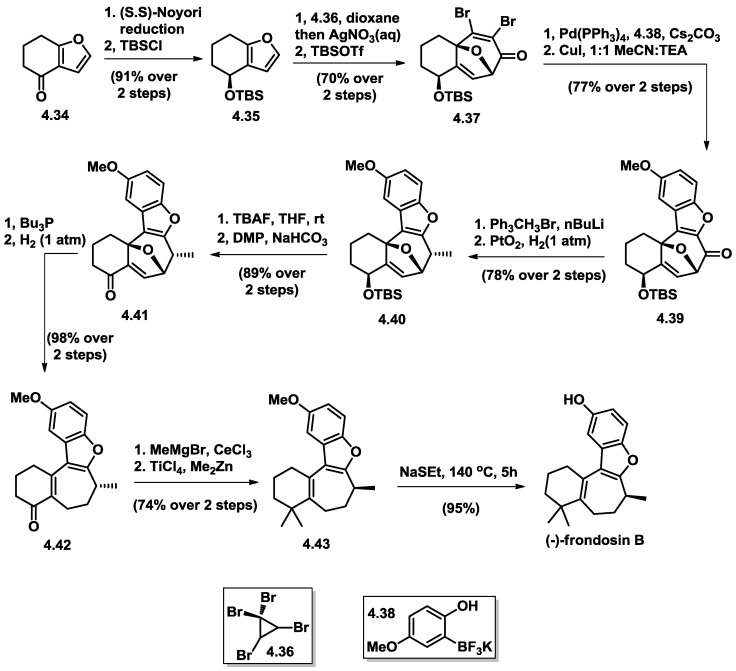
Wright’s total synthesis of (−)-frondosin B.

The Wright synthesis [30,31] was centered on a formal [4 + 3] cycloaddition reaction to generate the key 6,7-fused ring motif (Figure 13). This strategy was utilized on a collective total synthesis of (+)-liphagal and (−)-frondosin A as well.

The TBS-protected (*S*)-furyl-alcohol **4.35** was prepared with excellent enantioselectivity through reduction of ketone **4.34** with the (*S*,*S*)-Noyori catalyst mediated transfer hydrogenation, followed by TBSCl protection. The key cycloaddition reaction took place under aqueous phase with in situ generated bisbromocyclopropene from tetrabromocyclopropene (**4.36**). Regioselective Suzuki cross coupling between dibromo cycloheptenone (**4.37**) and aryl trifluoroborate salt (**4.38**) occurred exclusively at the β-bromide to deliver a phenol intermediate that triggered a Ullman-type coupling when treated with stoichiometric copper(I)iodide. Annulated benzofuran (**4.39)** was obtained in 77% yield over these two transformations. The regioselectivity of Suzuki coupling was attributed to electronic factors. Generation of C8 methyl group was operated through Wittig condensation gave hydrogenation from the exo-face, producing a single diastereomer (**4.40**). After cleavage of the silyl ether with TBAF, oxidation of the resulting allylic alcohol yielded enone **4.41** in 89% yield over two steps.

A Morrita–Balis–Hillman reaction effected with Bu_3_P led to oxy-bridge opening and generation of triene intermediate, which after regioselective hydrogenation produced enonen (**4.42**), a late-stage intermediate in the Trauner synthesis. Although it was pleasing that this enone showed spectroscopic data that matched those reported, the optical rotation for this intermediate surprisingly also matched that reported by Trauner. This result was disconcerting, because that compound was later shown to possess the incorrect S-configuration at C8, ultimately leading to the antipode of the natural product. The enone was advanced to (−)-frondosin B by Trauner’s protocol [26] and displayed the opposite rotation value to that reported for (+)-frondosin B ([α]_D_ = –16.7). It was straightforward to prepare the natural configuration from the antipode of silyoxy compound by utilizing the (*R*,*R*)-Noyori catalyst for the reduction of ketone produced the enantiomeric compound (after protection as its silyl ether), which could be taken through the same sequence to deliver (+)-frondosin B ([α]_D_ = +16.4).

Through synthesis of (+)- and (−)-frondosin B and related isotopic labeling studies, Wright and co-workers suggested an alternative mechanism for the isomerization, involving a ring contraction/expansion of a delocalized carbocation intermediate that ultimately led to inversion of the C8 center.

### 4.6. Synthetic Studies towards Frondosin B

Besides above six asymmetric total synthesis towards frondosin B, there are also several synthetic studies reported by Mehta [32], Flynn [33], and Winne [21] as well as the previously mentioned work [20]. There also were many synthetic efforts towards other frondosin members, including Trost’s total synthesis of (+)-frondosin A using Ru-catalyzed [5 + 2] cycloaddition [34,35]. An early review on synthesis of frondosins was summarized in 2011 [36] as well as in 2015 [31].

## 5. Conclusions

Since the isolation of these marine-derived meroterpenes, significant interest as well as efforts have been directed towards synthesizing (+)-liphagal, (+)-frondosin B, and related analogues. Among these total syntheses, biomimetic strategies and/or traditional disconnections and transformations were employed as summarized. However, in order to perform ideal total synthesis with the atom economy and steps economy, some new types of chemistry might be introduced and developed. C-C and C-H bond activation have been emerged as powerful synthetic tools over the last decades. As a matter of fact, synthetic studies towards the total synthesis of liphagal and frondosins using the C-C and C-H bond activation strategy are being conducted and will be reported in due course.
